# Three-Dimensional Virtual Anatomy as a New Approach for Medical Student’s Learning

**DOI:** 10.3390/ijerph182413247

**Published:** 2021-12-16

**Authors:** Anna Bartoletti-Stella, Valentina Gatta, Giulia Adalgisa Mariani, Pietro Gobbi, Mirella Falconi, Lucia Manzoli, Irene Faenza, Sara Salucci

**Affiliations:** 1 Department of Experimental, Diagnostic and Specialty Medicine (DIMES), University of Bologna, 40126 Bologna, Italy; anna.bartoletti2@unibo.it; 2 Cellular Signalling Laboratory, Department of Biomedical and NeuroMotor Sciences (DIBINEM), University of Bologna, 40126 Bologna, Italy; valentina.gatta6@unibo.it (V.G.); adalgisa.mariani@unibo.it (G.A.M.); mirella.falconi@unibo.it (M.F.); lucia.manzoli@unibo.it (L.M.); irene.faenza2@unibo.it (I.F.); 3 Department of Biomolecular Sciences (DiSB), Urbino University Carlo Bo, 61029 Urbino, Italy; pietro.gobbi@uniurb.it

**Keywords:** virtual gross anatomy, medical student learning, radiologist and surgical training, virtual cadaveric dissection, DICOM images

## Abstract

Most medical and health science schools adopt innovative tools to implement the teaching of anatomy to their undergraduate students. The increase in technological resources for educational purposes allows the use of virtual systems in the field of medicine, which can be considered decisive for improving anatomical knowledge, a requisite for safe and competent medical practice. Among these virtual tools, the Anatomage Table 7.0 represents, to date, a pivotal anatomical device for student education and training medical professionals. This review focuses attention on the potential of the Anatomage Table in the anatomical learning process and clinical practice by discussing these topics based on recent publication findings and describing their trends during the COVID-19 pandemic period. The reports documented a great interest in and a positive impact of the use of this technological table by medical students for teaching gross anatomy. Anatomage allows to describe, with accuracy and at high resolution, organ structure, vascularization, and innervation, as well as enables to familiarize with radiological images of real patients by improving knowledge in the radiological and surgical fields. Furthermore, its use can be considered strategic in a pandemic period, since it ensures, through an online platform, the continuation of anatomical and surgical training on dissecting cadavers.

## 1. Introduction

Human anatomy is a fundamental subject of study in the process of teaching and training medical students, since anatomical knowledge is a requisite for safe and competent medical practice [1,2,3,4,5], and it is indispensable in medical curricula.

Cadaveric dissection is a standardized approach essential to reaching a solid knowledge of anatomy and to know in situ anatomical abnormalities and variations [6,7]. Through dissection practice, students can orient themselves inside the human body to understand where the main topographical landmarks are localized and to describe anatomical three-dimensional (3D) relations. Therefore, dissection represents a huge advantage compared to single-dimensional images in textbooks, not only for students, but also for post-graduates and specialists. Dissection improves clinical training, and it is useful for surgeons who, through cadavers, can acquire greater safety and dexterity and can test deviceful surgical procedures [8,9]. Practice on human cadavers allows to develop medical procedure skills, for instance, its role in the learning of lung auscultation was described by Bakalarski and coworkers [10]. 

Thus, dissecting cadavers is the most relevant teaching program for studying human gross anatomy, and in our Institute of Human Anatomy of the Alma Mater Studiorum, University of Bologna, there is a consolidated tradition of using human body dissection for anatomical education [11].

However, the number of bodies available does not allow to meet the different requests, due to the growing interest in anatomical dissection exercises and the exponential increase in the number of students enrolled in medical degrees [12]. 

In recent years, different resources, such as prosections, plastination, computer-based learning, medical imaging, and living anatomy, have been considered to improve students’ anatomical learning. Estai and Bunt suggest that the use of plastination or projections appears suitable for the anatomical learning of students in dentistry, pharmacy, biology, nursing, and physical education [13]. On the other hand, the use of cadaveric dissection remains the best training resource for a future physician, which could be integrated by virtual dissecting devices [13,14]. Recently, virtual reality seems to play a crucial role as a new technology for enhancing education through new approaches for interactive student learning [14,15,16]. 

Different virtual reality systems have been proposed for anatomical learning, but they show several limitations in the study of gross anatomy, since their use is limited to specific anatomical topics [17].

More recently, a new teaching tool has been introduced in the academic world to improve and facilitate student learning. It is based on a new technology that allows to study human anatomy through virtual travel in three-dimensional human body models. The Anatomage Table 7.0 (Anatomage Inc., San Jose, CA, USA) is a virtual table through which one can observe human cadaver dissection in a 3D spatial dimension [18]. 

Four digitalized cadavers are loaded on the Anatomage Table 7.0: an Asian woman with a gastric cancer, an Asian man with acute leukemia, a Caucasian women with cardiac disease, and a Caucasian man with a brainstem tumor; all died from respiratory complications [19]. Consequently, through the use of these real dissecting cadavers, students can really know an organ’s size, position, vascularization, innervation, and relationship with the other organs. Furthermore, since these cadavers are affected by diseases, the observation of them allows students to improve their knowledge of not only organ variation, but also pathology. This last statement represents a very important source of clinical training, as well, which can be enhanced by the possibility to upload Digital Imaging and Communications in Medicine (DICOM), including radiological data. DICOM images allow students be confident in their approach to anatomical images, and students can compare them with the organ section of the human body in normal or pathological conditions, a very useful resource for surgical and radiological education [20,21,22]. Therefore, Anatomage is a virtual dissection table in which one can recognize gross anatomy components exactly as on a fresh cadaver [23]. It reconstructs 3D individual organs, which can be analyzed in all planes, dissectible in 3D and subject to an interactive segmentation. The exploration and learning of human anatomy [23,24] are certainly the strengths of the Anatomage Table, which are supported by its performed technology, which permits to show real, accurate anatomy, enough to be considered an innovative diagnostic tool in academic medical institutions.

For all these positive characteristics, the virtual table has been adopted as a complementary approach for practical anatomical exercises (without replacing the classic dissection exercises on real dissecting corpses) aimed at the students of the School of Medicine and Surgery of the University of Bologna [11].

The rationale of this review is to emphasize the adjunct value of the Anatomage Table as a complementary tool in the curriculum of a physician without forgetting that the dissection of cadavers remains, to date, the best practical resource, which the medical career cannot disregard. The main objective of this review is to highlight on the applications of the Anatomage Table based on recent literature data, and with particular regard to student learning within the clinical context and during a pandemic period, such as COVID-19.

## 2. Materials and Methods

The data were searched for and collected through multiple databases, including PubMed, Scopus, and Web of Science, considering the articles or reviews published in the last fifteen years. The key words used were: Anatomage Table, virtual device, anatomy learning, virtual tool and clinical practice, cadaveric dissection, COVID-19 and anatomy teaching, and lockdown and medical e-learning. About seventy articles were selected and included in the review. The results were then divided into three distinct sections.

## 3. Results

### 3.1. Anatomage for Student Learning

The Anatomage Table as part of gross anatomy courses in medical school and its positive outcome on student learning was demonstrated and described by several authors [23,24,25,26]. It was so successful that it was also used in dissection courses [18,27], where it replaced dissecting cadavers. In this regard, the study of the gross anatomy of the pelvis and musculoskeletal system via the Anatomage Table revealed that this modality appears equivalent to cadaveric dissection [2]. 

**Figure 1:**
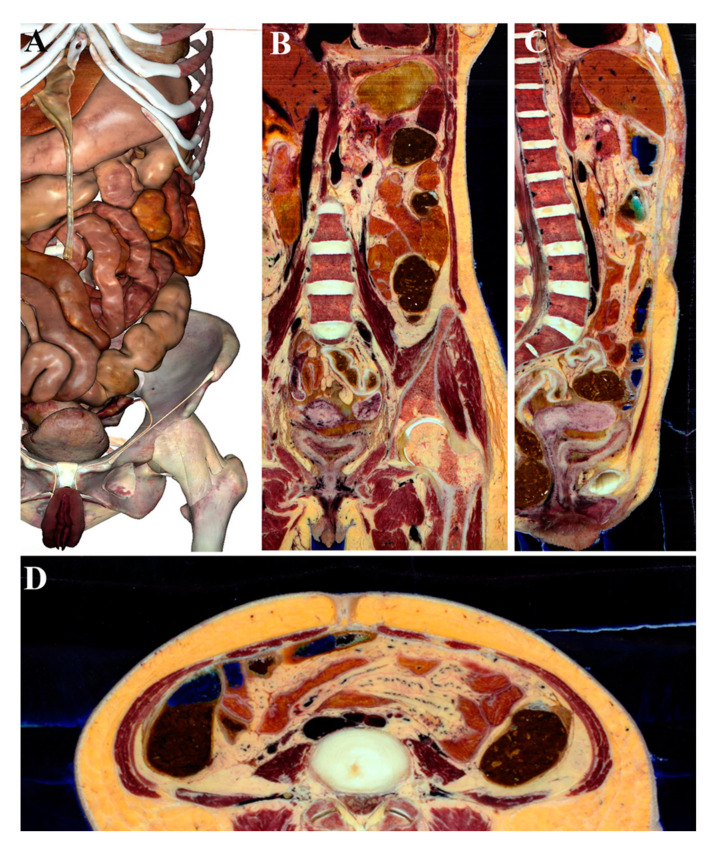
Abdominal region (**A**) of Asian adult male observed in the coronal (**B**), sagittal (**C**), and axial projection plane (**D**).

**Figure 2:**
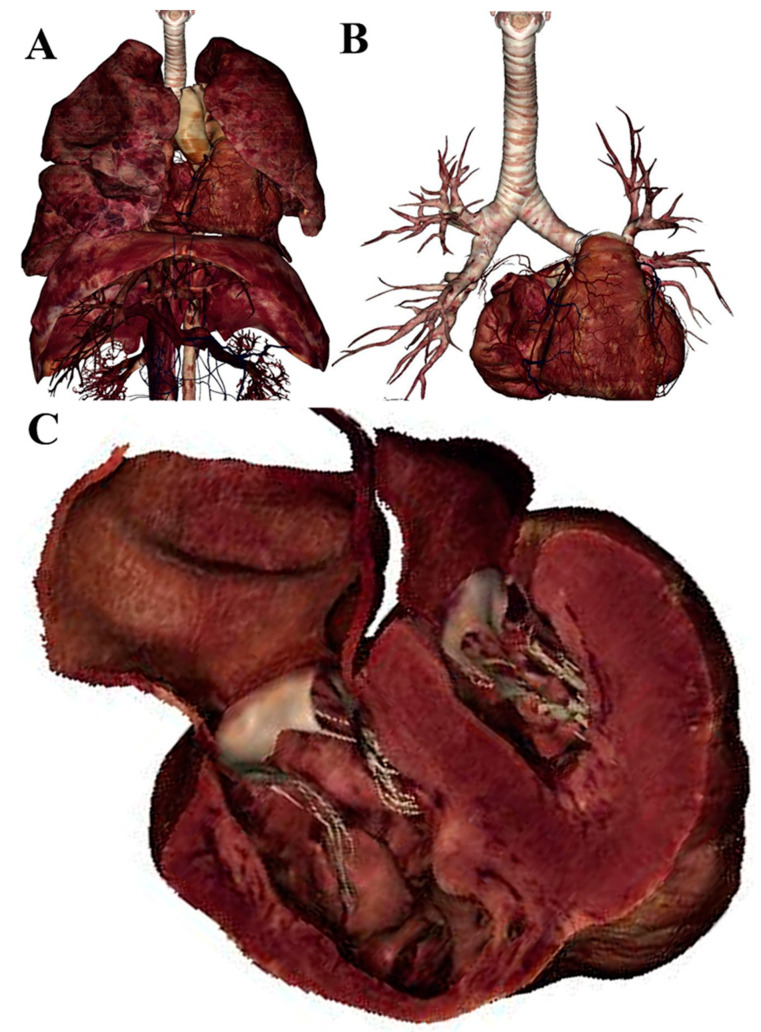
Thoracic region (**A**) of Asian adult man: lungs, heart, aorta, trachea, diaphragm, and the blood vessels of the abdominal region can be observed. The heart, trachea, and bronchial tree can be visualized, while the abdominal region vessels, lungs, diaphragm, aorta were deleted (**B**). In (**C**), a high magnification image of the heart without (a transversal cut was taken) the main vessels appears, in which it is possible clearly to distinguish the ventricular cardiac walls and the atrioventricular valves.

The literature data suggest that the Anatomage Table captured the enthusiasm of the students who are particularly predisposed to the use of technology in facilitating learning [28,29]. Medical students are enthusiastic about the possibility of studying male and female gross anatomy through human cadavers on a life-size touch screen table. Therefore, through the virtual table, students can dissect (all three section planes can be visualized, as shown in Figure 1), rotate, or zoom anatomical structures (Figure 2).

In particular, the users can isolate a single organ by deleting the structures with which this one is related: other organs, vessels, nerve, ligaments, muscles, and skin (Figure 2).

**Figure 3:**
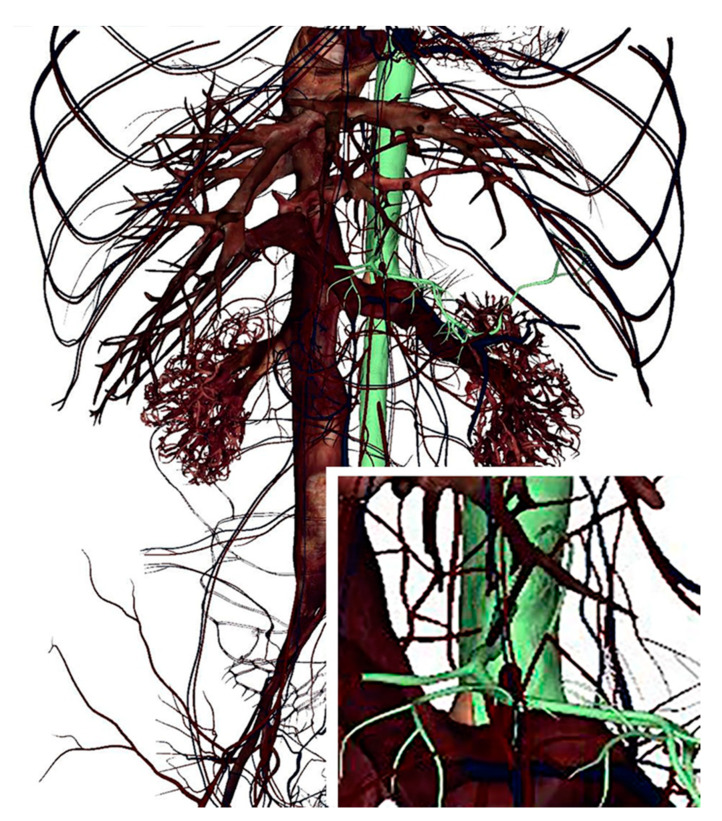
Vascularization of abdominal region. The abdominal aorta and the celiac trunk are green counterstained. In the inset, the celiac trunk can be observed at a higher magnification.

**Figure 4:**
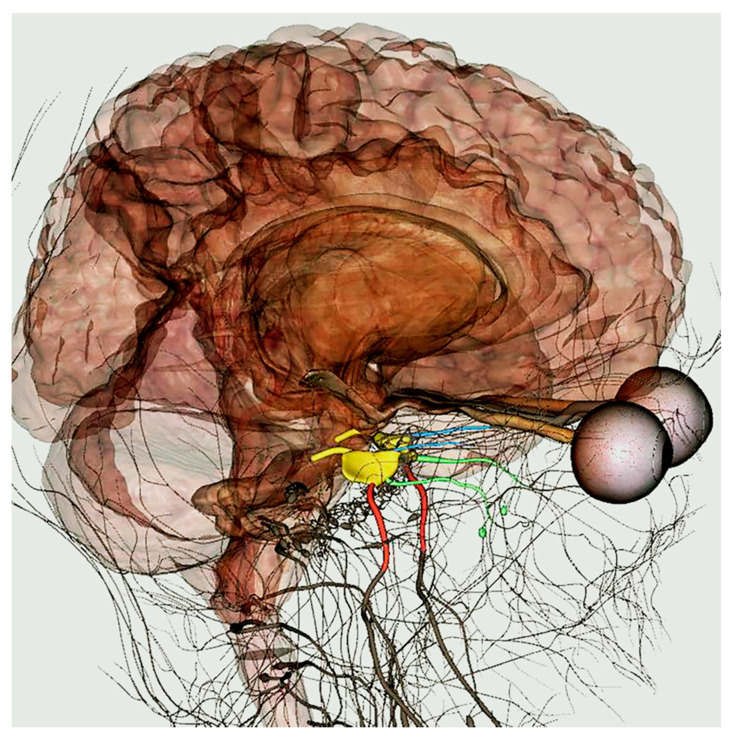
Trigeminus nerve with its semilunar ganglion (yellow) and its branches: mandibular (red), maxillary (green), and ophthalmic (light blue). To identify the origin of this nerve, the organs of the central nervous system were made transparent by appropriate filters.

Furthermore, medical students or clinicians, through different filters, can analyze specific structures, for instance, the vascular or innervation course of a specific anatomical district; in this regard, users can highlight with different colors a particular vessel or nerve and recognize it from the annotation (Figure 3 and Figure 4). Vascularization is perfectly described in the Anatomage cadavers, and the blood vessel course and its branches can be identified and distinguished, as shown in Figure 3, where, for instance, the abdominal aorta and celiac trunk can be observed.

However, the innervation should be improved, since, even if the main nerves (for instance, their origin or leak from a main hole) can be easily identified (Figure 4), nerve branches are not always present and often cannot be highlighted by filters, making them more difficult to localize by users.

Another criticism is correlated to the difficulty of identifying the connective fascial system that is absent in most anatomical regions.

Among the advantages of Anatomage, there is the possibility to observe radiological images [2] near to cadaveric projections. DICOM images are preinstalled onto the virtual system, including computed tomography and magnetic resonance, which, in our opinion, should be implemented to have an increasingly wide picture in the radiological field. Due to the presence of DICOM images, students or radiologists can switch from the cadaveric modality to the radiological one. This last table ability permits the users to familiarize themselves with a radiography of a full-sized human skeleton and understand the localization of the single bone components [28]. Moreover, it is possible to insert DICOM images of real patients, allowing the 3D reconstruction of the considered anatomical district and, thus, it is possible to study single cases and observe them on the LCD screen both in normal and pathological conditions.

For all these advantages, several reports demonstrated that Anatomage represents a fundamental teaching resource for anatomical learning, with the aim to be considered a complementary tool in supporting the medical education process [30,31,32]. Moreover, its use could appear essential for students in the clinical years of medical undergraduate training, which have already shown positive outcomes in reviewing gross anatomy (the learning of which is restricted to the first and second year of a medical academic track) through innovative methods, such as videoconferences or virtual tools [33].

One report demonstrated that the majority of medical students of Ethiopia University was particularly satisfied with learning medicine using this touch screen table [34]. This finding was then confirmed by Bin Abdulrahman and coworkers [35], who performed a cross-sectional study on medical students of the first year of Saud Islamic University, collecting a broad consensus on the use of the anatomical table for learning. Furthermore, this study suggests that the virtual reality-based computer technology is more effective than the traditional method in the learning of anatomy, since the users can understand in a 3D way the size, position, and relationships of organs, vessels, nerves, and muscles [35]. Therefore, Anatomage is an excellent virtual system with which to learn 3D realistic anatomy, allowing the exploration of, with high resolution, all anatomical districts, playing a crucial role not only for medical student education, but in the radiological and surgical fields, as well. Its use could help young medicals to understand different pathologies and to practice on a virtual cadaver. Thus, it represents an adjunct value for surgeons, who can observe and discuss surgical cases and can more easily propose the appropriate strategy. Finally, the Anatomage Table could represent a huge opportunity for other health disciplines with the aim to improve the knowledge of anatomy and to enhance the student’s long-term knowledge retention. In this regard, Narware and Neumeier described the success obtained from the use of virtual tables for nursing students at Canada University; for these students, cadaveric dissection is not expected [14].

### 3.2. Anatomage in Clinical Practice

Several data encourage the use of the Anatomage Table from students, who through this tool have the possibility to virtually dissect a digital, life-sized human cadaver and understand organ alterations [2,36]. Knowing the anatomical variations is crucial for medical student education [37]. Some variations are already easily detectable in dissecting cadavers, on organs such as the stomach and the brain. The loaded Asian female dissecting Asian female cadaver suffered from gastric cancer and presented respiratory impairment, conditions that caused her to die. These cadavers, however, may present other variations that are not immediate and that require a careful study of specific anatomical regions to be identified. Some researchers analyzed and studied the cadavers loaded on the Anatomage Table to investigate and describe their anatomical variations. For instance, the Caucasian male cadaver shows a rare alteration in the localization of the marginal mandibular nerve in the mandible region [38], which appears deeper than the facial vessels. Usually, this nerve, which is a branch of the facial nerve, runs superficially to the facial artery and vein in proximity of the inferior border of the mandible [38]. This rare alteration appears to be of great relevance and suggests particular attention in submandibular approaches to the neck and facial skeleton.

Furthermore, Panagouli and co-workers [19] demonstrated that the Asian female cadaver with gastric cancer has an unusual bilateral duplication of the suprascapular vein, which, usually crossing the back triangle of the neck, enters in the external jugular vein [39], and it is accompanied, along its course, by the suprascapular artery. Both blood vessels are localized over superior transverse scapular ligaments which form a foramen, in which pass the supravascular nerve. The Asian female cadaver shows two supravascular vein branches which drain in the subclavian vein. One branch is localized together with the scapular artery over the scapular ligaments; the other one passes under the same ligaments, accompanied by the supravascular nerve [19].

More recently, Al-Redouan and Kachilik [40], analyzing the supravascular vein alteration of the same female cadaver, suggested that the variation identified by Panaugouli and collaborators is not unusual, but rather common. It is, therefore, important to underline and identify the anatomical variations that characterize the cadavers loaded on the virtual table, since these human bodies are accessible all over the world [40], and anatomical variation discovery can reveal new scenarios to anatomical knowledge, which is useful for preventing surgical injuries during procedures.

Another application of the Anatomage Table is maxillofacial surgery, the knowledge of which is crucial for the assessment of several benign and malignant conditions of the head and neck districts. Similarly, medicals observed an odontogenic keratocyst by means of the Anatomage Table. This neck lesion was easily detected by students, who could appreciate and study it through different cuts (sagittal, axial, and coronal) and, consequently, investigate and explain the proposed surgical intervention [36]. Moreover, Brucoli and collaborators [20] loaded DICOM images of unilateral orbital floor fractures on Anatomage. This allowed young surgeons to recognize with higher definiteness anatomical dissection images in comparison with computed tomography scans, both in the preoperative and postoperative stages [23].

Furthermore, one study detailed the utility of the Anatomage Table to carefully identify an extranodal extension of a cervical lymph node metastasis [41]; the authors demonstrated that the Anatomage images showed a higher percentage of similarity with histopathological examinations than the computed tomography scans and magnetic resonance images [41].

The Anatomage Table can be considered a complementary system in implantology practice, as well. Three-dimensional cone-beam computed tomographies of jaws were converted into DICOM format and can be observed on Anatomage, where it is possible to measure the bone height and thickness where implants were necessary. Thus, alveolar bone morphological characteristics and anatomical variations (nasal fossa, mandibular canal, mental foramen, and sinuses) could be carefully detected, which allowed to specialize the surgical implant [42].

Furthermore, the Anatomage Table could find application in the study of morphological parameters according to sex or race, with potential application in anthropological or anthropometric studies. In particular, morphometric analyses can be carried out, and they appear useful to define somatic characteristics of a specific population. One study analyzed, through the virtual table, the temporomandibular joint in Chinese adults with normal occlusion and harmonious skeletons. The data obtained revealed a wide inter-fossa and inter-condyle distance and the existence of asymmetries between the right and left mandibular condyles: morphometric parameters which the authors demonstrated to be characteristic of the Chinese population [43].

### 3.3. Anatomage Table Utility during the COVID-19 Pandemic

The coronavirus SARS-CoV-2 infection represents a serious epidemic disease that causes human death by inducing a severe, atypical pneumonia known as acute respiratory distress syndrome. This symptomatology can also be characterized by some systemic complications, which can include dysfunctions in the cardiovascular [44,45], renal [46,47], hepatic [48,49], gastrointestinal [50,51], and central nervous [52,53] systems. This latest coronavirus disease, called COVID-19, started in Wuhan, China, and diffused in the world because of its high virulence [54]. Moreover, its transmissibility is very fast and happens primarily through aerosol droplets [44,54]. Being such a contagious virus, it has forced people into lockdown periods, compromising medical education and limiting the access to practical exercises.

However, some pedagogical practices are indispensable to reach a complete knowledge of anatomy [8,29]. One of these is dissection [8], which, as reported above, is recognized as an exclusive and necessary teaching and learning practice of medical curricula. Therefore, it would be unthinkable to interrupt medical training during a long pandemic. In this regard, technological resources can be considered a valid alternative for the teaching of subjects, such as histology, anatomy, and physiology, that are essential for a medical career [55]. The COVID-19 pandemic has remarkably influenced teaching and learning activities across the world, where medical students followed their anatomical lessons through online systems, and, as a consequence, in most academic medical schools, dissection has taken a backseat [56]. In this scenario, the Anatomage Table could represent a valid resource that allows, through an online platform, to learn dissecting gross anatomy during a pandemic period. In this regard, a study demonstrated its crucial role in discussing plastic surgery topics during the COVID-19 lockdown. During the pandemic, a reduced number of surgical interventions have been performed, and the Anatomage Table allowed the continuation of surgical training activities, since it can be combined with a software called Touch Surgery, available on tablets or smartphones [57].

This potentiality of Anatomage in achieving learning objectives during the lockdown is supported by another study performed on medical students of the Africa School [58]. Here, the virtual table was deployed to facilitate the effective teaching of anatomy and related basic medical science. Both studies [57,58] suggest that technological investment in Anatomage appears to be an important strategy for academic schools or institutions, which could easily adapt to alternative learning models during emergencies. However, even if these anatomical exercises based on distance learning appear valuable in a pandemic period, we must not forget that a physician should develop important virtues, such as courage, empathy, and compassion, which one can only learn through attendance of an anatomy laboratory [59]. Moreover, the use of educational virtual platforms, such as online lectures, prerecorded lectures, and prerecorded laboratory dissection videos, highlighted a certain degree of dissatisfaction of medical students, who have also showed a decline in educational performance, demonstrating how in-person teaching is still fundamental for medical careers [60]. On the other hand, in the Edimburg University, the experiences of teaching anatomy online, documented by Kelsey and coworkers, collected a positive consensus as an interesting and valuable experience [61]. Therefore, to date, there are controversial opinions on e-learning in anatomy. Certainly, the Anatomage Table, in our opinion, became an important device, useful in recognizing online 3D anatomical structures and their localization, during the COVID-19 lockdown, when dissection practice sessions were suspended or reduced.

Furthermore, the Anatomage Table proved to be useful in the diagnosis of COVID-19. To date, the detection of nucleic acid in nasal and throat swab samples is the diagnostic method for COVID-19 detection, and its confirmation is based on RT-PCR (real-time polymerase chain reaction) with a sensitivity of 75% and 95% for both sampling procedures [62]. Nevertheless, RT-PCR diagnosis showed some limitations due to sensitivity, sample transportation, the time required for gene amplification, and other factors. Some researchers then demonstrated that computed tomography scans without the injection of the contrast agent allow a rapid screening of the virus with a high sensitivity, about 98% [63,64]. Therefore, computed tomography scans could represent an alternative method in the early detection of COVID-19 pulmonary manifestations [64]. The lack of knowledge about the effect of the virus in the various organs, in particular the lungs, stimulated researchers to improve the 2D computed tomography scan, reconstructing it in 3D through the Anatomage Table. The authors performed this 3D reconstruction, comparing patients with a negative RT-PCR and normal computed tomography images with those that presented a positive RT-PCR and abnormal scan images. Anatomage allowed to obtain 3D unventilated lung volumes that appeared reliable and accurate for the diagnosis. In this way, Hasni and coworkers [62] showed that in COVID-19 patients, it is possible to distinguish the virus manifestations and the stage of disease, and propose the appropriate protocol. Moreover, the virtual table allowed to evaluate the therapeutic protocol efficacy through the comparison between 3D reconstructions of RT-PCR-positive patients before and after pharmacological treatment [62].

## 4. Conclusions

**Figure 5:**
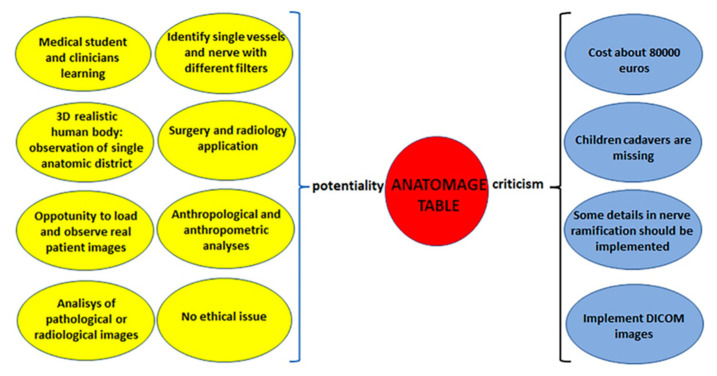
Anatomage Table strengths (yellow) vs. weak points (light blue).

The data analyzed so far show that the virtual table has numerous strengths that exceed the critical issues of the instrument, as schematized in Figure 5.

The real limit of Anatomage is related to the high cost due to restrictive licenses and expensive hardware, and another important point of weakness is the lack of children cadavers, which restricts application fields by excluding pediatric training. Its use exceeds the ethical and bureaucratic limits on which the cadaveric dissection depends. Dissection is characterized by some limitations in the human body donor program, and to ensure the autonomy of these potential cadaver donors, an adequate informed consent process must be carried out for ensuring that the bequest is a completely voluntary-based decision [11]. In addition, dissection on cadavers is considered, at first glance, a stressful experience that causes a wide range of symptoms among medical students, such as difficulty breathing, palpitations, and nausea [65,66]. These symptoms are associated with the strong odor caused by chemical substances used in the prevention of tissue decay and with the aim to kill potential pathogens [67]. All these limitations can be overcome using the virtual table, which can be considered a complementary device to dissection practice.

Therefore, these findings underline that the Anatomage Table plays a crucial role in the Medical Academy as a user-friendly touch screen table, extremely useful for medical students to learn gross human anatomy and to acquire familiarity with surgical approaches [68,69]. It is a potential tool for understanding organ variation during normal and pathological conditions [7]. It is essential for the educational training of students and clinicians, since 3D reconstruction proved its great interest in disease diagnosis, prognosis, and pedagogy, as demonstrated in the 3D reconstruction of the lungs [62]. Finally, this learning technology could result crucial during exceptional circumstances, such as pandemics, since, even if remotely, it allows to implement the teaching of anatomy on virtual dissecting cadavers, with positive outcomes in anatomical knowledge and in clinical practice.

## Data Availability

Not applicable.

## References

[1] Turney B.W. (2007). Anatomy in a Modern Medical Curriculum. Ann. R. Coll. Surg. Engl..

[2] Baratz G., Wilson-Delfosse A.L., Singelyn B.M., Allan K.C., Rieth G.E., Ratnaparkhi R., Jenks B.P., Carlton C., Freeman B.K., Wish-Baratz S. (2019). Evaluating the Anatomage Table Compared to Cadaveric Dissection as a Learning Modality for Gross Anatomy. Med. Sci. Educ..

[3] Kumar R., Singh R. (2020). Model pedagogy of human anatomy in medical education. Surg. Radiol. Anat..

[4] Saverino D. (2021). Teaching anatomy at the time of COVID-19. Clin. Anat..

[5] Thompson A.R., Giffin B.F. (2021). Higher-Order Assessment in Gross Anatomy: A Comparison of Performance on Higher- versus Lower-Order Anatomy Questions between Undergraduate and First-Year Medical Students. Anat. Sci. Educ..

[6] Kraszpulska B., Bomkamp D., Brueckner-Collins J. (2013). Benefits of Traditional Cadaveric Dissection in a Digital World: Medical and Dental Students’Perspectives. Med. Sci. Educ..

[7] Parker E., Randall V. (2020). Learning beyond the Basics of Cadaveric Dissection: A Qualitative Analysis of Non-academic Learning in Anatomy Education. Med. Sci. Educ..

[8] Ghosh S.K. (2017). Cadaveric dissection as an educational tool for anatomical sciences in the 21st century. Anat. Sci. Educ..

[9] Koop C.F.A., Marschollek M., Schmiedl A., Proskynitopoulos P.J., Behrends M. (2021). Does an Audiovisual Dissection Manual Improve Medical Students’ Learning in the Gross Anatomy Dissection Course?. Anat. Sci. Educ..

[10] Bakalarski P., Klepacka M., Sówka K., Boyko I., Głowala D., Bodecot B., Pinet Peralta L.M. (2019). Cadaver as a didactic tool for auscultating lung sounds. Crit. Care Innov..

[11] Orsini E., Quaranta M., Ratti S., Mariani G.A., Mongiorgi S., Billi A.M., Manzoli L. (2021). The whole body donation program at the university of Bologna: A report based on the experience of one of the oldest university in Western world. Ann. Anat..

[12] Boscolo-Berto R., Tortorella C., Porzionato A., Stecco C., Picardi E.E.E., Macchi V., De Caro R. (2021). The additional role of virtual to traditional dissection in teaching anatomy: A randomised controlled trial. Surg. Radiol. Anat..

[13] Estai M., Bunt S. (2016). Best teaching practices in anatomy education: A critical review. Ann. Anat..

[14] Narnaware Y., Neumeier M. (2021). Use of a virtual human cadaver to improve knowledge of human anatomy in nursing students: Research article. Teach. Learn. Nurs..

[15] Cheng K.H., Tsai C.C. (2013). Affordances of Augmented Reality in Science Learning: Suggestions for Future Research. J. Sci. Educ. Technol..

[16] Akçayır M., Akçayır G. (2017). Advantages and challenges associated with augmented reality for education: A systematic review of the literature. Educ. Res. Rev..

[17] Preim B., Saalfeld P. (2018). A survey of virtual human anatomy education systems. Comput. Graph..

[18] Fyfe S., Fyfe G., Dye D., Radley-Crabb H. (2018). The Anatomage table: Differences in student ratings between initial implementation and established use. Focus Health Prof. Educ. Multi-Prof. J..

[19] Panagouli E., Tsirigoti A., Kotsira G., Demesticha T., Skandalakis P., Troupis T., Filippou D. (2019). An Unusual Bilateral Duplication of the Suprascapular Vein and Its Relation to the Superior Transverse Scapular Ligament Revealed by Anatomage Table. Acta Med. Acad..

[20] Custer T.M., Michael K. (2015). The utilization of the Anatomage virtual dissection table in the education of imaging science students. J. Tomogr. Simul..

[21] Paech D., Giesel F.L., Unterhinninghofen R., Schlemmer H.-P., Kuner T., Doll S. (2017). Cadaver-specific CT scans visualized at the dissection table combined with virtual dissection tables improve learning performance in general gross anatomy. Eur. Radiol..

[22] Chew F.S., Relyea-Chew A., Ochoa E.R. (2006). Postmortem Computed Tomography of Cadavers Embalmed for Use in Teaching Gross Anatomy. J. Comput. Assist. Tomogr..

[23] Brucoli M., Boccafoschi F., Boffano P., Broccardo E., Benech A. (2018). The Anatomage Table and the placement of titanium mesh for the management of orbital floor fractures. Oral Surg. Oral Med. Oral Pathol. Oral Radiol..

[24] Eickmeyer S., Wertsch J., Lewandowski L., Hoagland T., Braza D. (2013). Teaching pelvic floor musculoskeletal anatomy using Anatomage. Clin. Anat..

[25] Brown J., Stonelake S., Anderson W., Abdulla M., Toms C., Farfus A., Wilton J. (2015). Medical student perception of anatomage—A 3D interactive anatomy dissection table. Int. J. Surg..

[26] Lewandowski L., Wertsch J., Hoagland T., Braza D. (2013). Exploration of Anatomage interfaced with ECHO 360 software. Clin. Anat..

[27] Chung B.S., Shin D.S., Brown P., Choi J., Chung M.S. (2015). Virtual Dissection Table Including the Visible Korean Images, Complemented by Free Software of the Same Data. Int. J. Morphol..

[28] Bork F., Stratmann L., Enssle S., Eck U., Navab N., Waschke J., Kugelmann D. (2019). The Benefits of an Augmented Reality Magic Mirror System for Integrated Radiology Teaching in Gross Anatomy. Anat. Sci. Educ..

[29] DiLullo C., McGee P., Kriebel R.M. (2011). Demystifying the Millennial student: A reassessment in measures of character and engagement in professional education. Anat. Sci. Educ..

[30] Ghosh S.K. (2020). Transformation of the role of human dissection in medical education: Cultivating principles of medical ethics. Surg. Radiol. Anat..

[31] Ward T.M., Wertz C.I., Mickelsen W. (2018). Anatomage Table Enhances Radiologic Technology Education. Radiol. Technol..

[32] Bhandari K., Acharya S., Srivastava A.K., Kumari R., Nimmagada H.K. (2016). Plastination: A new model of teaching anatomy. Int. J. Anat. Res..

[33] Allsop S., Hollifield M., Huppler L., Baumgardt D., Ryan D., Eker M., Spear M., Fuller C. (2020). Using videoconferencing to deliver anatomy teaching to medical students on clinical placements. Transl. Res. Anat..

[34] Alfawzan A., Alfawzan O., Alessa R., Alturki A., Alshiha K., Omair A., Agha S., Mahzari M. (2020). An assessment of learning styles among undergraduate medical students at King Saud Bin Abdulaziz University (KSAUHS), King Saud University (KSU) and Imam Mohammad Ibn Saud Islamic University (IMSIU). Res. Sq..

[35] Bin Abdulrahman K.A., Jumaa M.I., Hanafy S.M., Elkordy E.A., Arafa M.A., Ahmad T., Rasheed S. (2021). Students’ Perceptions and Attitudes After Exposure to Three Different Instructional Strategies in Applied Anatomy. Adv. Med. Educ. Pract..

[36] Brucoli M., Boffano P., Pezzana A., Sedran L., Boccafoschi F., Benech A. (2020). The potentialities of the Anatomage Table for head and neck pathology: Medical education and informed consent. Oral Maxillofac. Surg..

[37] Alraddadi A. (2021). Literature Review of Anatomical Variations: Clinical Significance, Identification Approach, and Teaching Strategies. Cureus.

[38] Strantzias P., Botou A., Manoli A., Skandalakis P.N., Filippou D. (2019). Variation of Marginal Mandibular Nerve in a Caucasian Male Cadaver: A Study Using the Anatomage Table. Cureus.

[39] Standring S., Borley N.R., Collins P., Crossman A.R., Gatzoulis M.A., Healy J.C., Johnson D., Mahadevan V., Newell R.I.M., Wigley C.B. (2008). Gray’s Anatomy. The Anatomical Basis of Clinical Practice.

[40] Al-Redouan A., Kachlik D. (2020). Letter to the Editor: Regarding “An Unusual Bilateral Duplication of the Suprascapular Vein and Its Relation to the Superior Transverse Scapular Ligament Revealed by Anatomage Table”. Acta Med. Acad..

[41] Tirelli G., de Groodt J., Sia E., Belgrano M.G., Degrassi F., Boscolo-Rizzo P., Cova M.A., Marcuzzo A.V. (2021). Accuracy of the Anatomage Table in detecting extranodal extension in head and neck cancer: A pilot study. J. Med. Imaging.

[42] Kurt Bayrakdar S., Orhan K., Bayrakdar I.S., Bilgir E., Ezhov M., Gusarev M., Shumilov E. (2021). A deep learning approach for dental implant planning in cone-beam computed tomography images. BMC Med. Imaging.

[43] Abdulqader A.A., Ren L., Alhammadi M., Abdu Z.A., Mohamed A.A.S. (2020). Three-dimensional analysis of temporomandibular joint in Chinese adults with normal occlusion and harmonious skeleton. Oral Radiol..

[44] Zheng Y.-Y., Ma Y.-T., Zhang J.-Y., Xie X. (2020). COVID-19 and the cardiovascular system. Nat. Rev. Cardiol..

[45] Tajbakhsh A., Gheibi Hayat S.M., Taghizadeh H., Akbari A., Inabadi M., Savardashtaki A., Johnston T.P., Sahebkar A. (2021). COVID-19 and cardiac injury: Clinical manifestations, biomarkers, mechanisms, diagnosis, treatment, and follow up. Expert Rev. Anti Infect. Ther..

[46] Ronco C., Reis T. (2020). Kidney involvement in COVID-19 and rationale for extracorporeal therapies. Nat. Rev. Nephrol..

[47] Ertuğlu L.A., Kanbay A., Afşar B., Elsürer Afşar R., Kanbay M. (2020). COVID-19 and acute kidney injury. Tuberk. Toraks.

[48] Zhang C., Shi L., Wang F.-S. (2020). Liver injury in COVID-19: Management and challenges. Lancet Gastroenterol. Hepatol..

[49] Tian D., Ye Q. (2020). Hepatic complications of COVID-19 and its treatment. J. Med. Virol..

[50] Yeo C., Kaushal S., Yeo D. (2020). Enteric involvement of coronaviruses: Is faecal–oral transmission of SARS-CoV-2 possible?. Lancet Gastroenterol. Hepatol..

[51] Al Argan R.J., Alqatari S.G., Al Said A.H., Alsulaiman R.M., Noor A., Al Sheekh L.A., Al Beladi F.H. (2021). Gastrointestinal perforation secondary to COVID-19: Case Reports and Literature Review. Medicine.

[52] Baig A.M., Khaleeq A., Ali U., Syeda H. (2020). Evidence of the COVID-19 Virus Targeting the CNS: Tissue Distribution, Host–Virus Interaction, and Proposed Neurotropic Mechanisms. ACS Chem. Neurosci..

[53] Carod-Artal F.J. (2020). Neurological complications of coronavirus and COVID-19. Rev. Neurol..

[54] Salimi S., Hamlyn J.M. (2020). COVID-19 and Crosstalk With the Hallmarks of Aging. J. Gerontol. A. Biol. Sci. Med. Sci..

[55] Cheng X., Chan L.K., Cai H., Zhou D., Yang X. (2021). Adaptions and perceptions on histology and embryology teaching practice in China during the Covid-19 pandemic. Transl. Res. Anat..

[56] Longhurst G.J., Stone D.M., Dulohery K., Scully D., Campbell T., Smith C.F. (2020). Strength, Weakness, Opportunity, Threat (SWOT) Analysis of the Adaptations to Anatomical Education in the United Kingdom and Republic of Ireland in Response to the Covid-19 Pandemic. Anat. Sci. Educ..

[57] Zingaretti N., Contessi Negrini F., Tel A., Tresoldi M.M., Bresadola V., Parodi P.C. (2020). The Impact of COVID-19 on Plastic Surgery Residency Training. Aesthet. Plast. Surg..

[58] Owolabi J., Bekele A. (2021). Implementation of Innovative Educational Technologies in Teaching of Anatomy and Basic Medical Sciences During the COVID-19 Pandemic in a Developing Country: The COVID-19 Silver Lining?. Adv. Med. Educ. Pract..

[59] Pearson S. (2020). Anatomy: Beyond the COVID-19 pandemic. Acad. Med..

[60] Flynn W., Kumar N., Donovan R., Jones M., Vickerton P. (2021). Delivering online alternatives to the anatomy laboratory: Early experience during the COVID-19 pandemic. Clin. Anat..

[61] Kelsey A.H.C.M., McCulloch V., Gillingwater T.H., Findlater G.S., Paxton J.Z. (2020). Anatomical sciences at the University of Edinburgh: Initial experiences of teaching anatomy online. Transl. Res. Anat..

[62] Hasni M., Farahat Z., Abdeljelil A., Marzouki K., Aoudad M., Tlemsani Z., Megdiche K., Ngote N. (2020). An efficient approach based on 3D reconstruction of CT scan to improve the management and monitoring of COVID-19 patients. Heliyon.

[63] Fang Y., Zhang H., Xie J., Lin M., Ying L., Pang P., Ji W. (2020). Sensitivity of Chest CT for COVID-19: Comparison to RT-PCR. Radiology.

[64] Kohli A. (2020). Can imaging impact the coronavirus pandemic?. Indian J. Radiol. Imaging.

[65] Chia T.I., Oyeniran O.I., Ajagbe A.O., Onigbinde O.A., Oraebosi M.I. (2020). The symptoms and stress experienced by medical students in anatomy dissection halls. J. Taibah Univ. Med. Sci..

[66] Zubair A., Waheed S., Shuja F. (2021). Psychological impact of cadaveric dissection on first-year medical students. J. R. Coll. Physicians Edinb..

[67] Aung W.Y., Sakamoto H., Sato A., Yi E.E., Thein Z.L., Nwe M.S., Shein N., Linn H., Uchiyama S., Kunugita N. (2021). Indoor Formaldehyde Concentration, Personal Formaldehyde Exposure and Clinical Symptoms during Anatomy Dissection Sessions, University of Medicine 1, Yangon. Int. J. Environ. Res. Public Health.

[68] Tenaw B. (2020). Teaching gross anatomy: Anatomage table as an innovative line of attack. Int. J. Anat. Var..

[69] Yammine K., Violato C. (2015). A meta-analysis of the educational effectiveness of three-dimensional visualization technologies in teaching anatomy. Anat. Sci. Educ..

